# Association of cholesterol and glycemic state biomarkers with phenotypic variation and Parkinson's disease progression: The Oxford Discovery cohort

**DOI:** 10.1177/1877718X251323914

**Published:** 2025-04-13

**Authors:** Nicholas Kwan Ho Chiu, Michael Lawton, Tanja Zerenner, Alireza Morovat, Jessica Welch, Jamil Razzaque, Michele T Hu, Yoav Ben-Shlomo

**Affiliations:** 1 Margaret K. L. Cheung Research Centre for Management of Parkinsonism, Department of Medicine and Therapeutics, Faculty of Medicine, The Chinese University of Hong Kong, Hong Kong, China; 2 Oxford Parkinson's Disease Centre, University of Oxford, Oxford, UK; 3 Population Health Sciences, Bristol Medical School, University of Bristol, Bristol, UK; 4 Department of Clinical Biochemistry, Oxford University Hospital NHS Foundation Trust, Oxford, UK; 5 Nuffield Department of Clinical Neurosciences, Division of Clinical Neurology, University of Oxford, Oxford, UK; 6 Department of Clinical Neurology, Oxford University Hospital NHS Foundation Trust, Oxford, UK

**Keywords:** HDL-C, cholesterol, fructosamine, Parkinson’s disease, biomarkers, prognosis

## Abstract

**Background:**

Parkinson's disease (PD) has marked phenotypic variability. Increased lipids have been suggested as being neuroprotective whilst hyperglycemia may increase α-synuclein aggregation.

**Objective:**

We have tested whether high total cholesterol and high-density lipoprotein cholesterol (HDL-C) and low levels of fructosamine are associated with better PD phenotypes and predict less rapid progression

**Methods:**

Non-fasting serum HDL-C, total cholesterol, and fructosamine were measured at baseline in 866 patients with early PD (median duration, 0.96; IQR, 0.43–1.98 years) from the Oxford Discovery cohort. These biomarkers were compared against our data-derived PD subtypes using multinomial logistic regression. We used multilevel models to predict longitudinal motor and non-motor outcomes (e.g., cognition, mood).

**Results:**

HDL-C and total cholesterol differed across baseline PD phenotype clusters, with reduced levels associated with the most severe motor and non-motor phenotypes (psychological well-being, cognitive impairment, REM sleep behavior disorder, and daytime sleepiness). Higher HDL-C and total cholesterol, although the latter was attenuated after adjustment for statin use, were associated with better baseline activities of daily living (e.g., UPDRS-II score with 1 SD increase in HDL-C −0.74, 95%CI −1.22 to −0.26, p = 0.002) and non-motor features. Neither predicted the rate of motor or non-motor progression. Fructosamine levels were not associated with phenotypic variability or rate of disease progression.

**Conclusions:**

Hypercholesterolemia was associated with a better motor/non-motor disease subtype and daily living impairment at presentation, but did not predict longitudinal change. Future research needs to determine if these associations are causally related or secondary to disease onset by examining prodromal subjects.

## Plain language summary

People with Parkinson's disease (PD) can have quite different features when they first present and may get worse at different rates. We wanted to test whether the levels of fats and sugar control predicted worse presentation and differences in how the disease develops, as had been suggested by past research. We used blood tests for cholesterol (total and HDL cholesterol) and fructosamine (a measure of glucose control) from 866 patients with early PD (half within a year of diagnosis) from the Oxford Discovery cohort. We used sophisticated statistical models to test if the blood levels predicted changes in motor and non-motor outcomes (e.g., cognition, mood) over time. We found lower levels of HDL and total cholesterol were associated with the most severe motor and non-motor phenotypes (psychological well-being, cognitive impairment, REM sleep behavior disorder, and daytime sleepiness). Higher HDL cholesterol was associated with slightly better baseline activities of daily living. Neither, however, predicted the rate of motor or non-motor progression. A higher fructosamine (worse control of sugar) also did not predict changes in motor function. Our results suggest that higher cholesterol is associated with a better motor/non-motor disease subtype and slight benefits in daily living impairment at presentation but does not predict longitudinal change in contrast to past research. Future research needs to determine if these associations in early disease have a causal role or are secondary to disease onset by examining subjects who are at high risk of developing PD in the future.

## Introduction

Parkinson's disease (PD) is a neurodegenerative disease with significant heterogeneity in motor and non-motor phenotype at presentation. Studies in early PD have shown marked differences in disease progression in terms of cognitive and motor decline over time.^1^ This variability may contribute to inconsistent responses to disease-modifying therapy in clinical trials. Identifying simple and affordable prognostic markers that predict disease progression would facilitate trial designs that target patients with more aggressive disease progression.

Vascular risk factors such as diabetes mellitus and hypercholesterolemia have been shown to be associated with an increase in PD incidence and predict a worse progression of motor and non-motor symptoms including cognitive functioning in PD.^2–6^ Studies have suggested that higher high-density lipoprotein cholesterol (HDL-C) may have a neuroprotective role in the development of PD, although the evidence is limited and controversial.^2,6^ Low apolipoprotein A-I (apo A-I), a major component of HDL-C, has been shown to correlate with an earlier onset, more severe motor impairment, and more severe disease phenotype.^7^ However, the DeNoPa trial found that higher levels of baseline HDL-C predicted worse cognitive progression in early PD.^5^

Higher serum total cholesterol (TC) levels has been associated with a decreased risk of PD development, possibly through a role in synapse repair and anti-oxidant effects through coenzyme Q_10_.^8,9^ However, the associations of TC with clinical features are confusing. Lower levels have also been associated with increased non-motor impairment including mood disturbances in PD.^10,11^ To our knowledge, the DATATOP trial is the only study providing evidence that higher baseline TC may be associated with slower clinical progression in PD by measuring time to endpoint of disability warranting dopaminergic therapy.^12^ To further complicate the picture, statins, which lower TC, have been associated with both an increased and decreased risk of PD, although these may reflect confounding by lipid levels or other methodological biases.^13^

Diabetes mellitus has been shown to be a risk factor for PD,^14,15^ and is associated with worse motor function and depressive symptoms.^3,16^ It may also accelerate progression of motor and cognitive decline in PD.^17,18^ Although HbA1c offers the best clinically-useful assessment of glycemic status, it requires whole blood for analysis. Fructosamine, a measure of glycated serum proteins, is a stable biomarker of glycemic state over the preceding few weeks^19^ and offers a reasonable means of assessing stored serum specimens when whole blood is unavailable. Fructosamine may reflect the detrimental effects of protein glycation, leading to formation of advance glycation end products, shown to induce aggregation and toxicity of α-synuclein.^20^ It would be reasonable to hypothesize that a higher fructosamine would predict a worse PD progression, even though there is limited evidence for this.

We have tested the following *a priori* hypotheses that high TC and HDL-C but low levels of fructosamine are associated with better PD phenotypes in early disease and will predict less rapid motor and non-motor progression using the Oxford Discovery cohort study, one of the best characterized and largest longitudinal study of PD to see if we replicate, or not, previously reported associations.

## Methods

### Participants

We selected 866 PD patients (64.3% male; median disease duration, 0.96 years; IQR, 0.43–1.98) with serum samples within 3.5 years of diagnosis as part of the Oxford Parkinson's Disease Centre Discovery cohort study. Participants were recruited between September 2010 and January 2015 from 11 hospitals in the Thames Valley, covering a population of approximately 2.1 million. All subjects had a probability of PD ≥ 90% as rated by a movement disorder specialist or a PD research neurologist based on their latest assessments. They were followed up every 18 months at our research clinics, but individuals who could not attend in person were monitored with telephone follow-up instead.

### Ethics approval

This study received NRES ethical approval from the South Central Oxford A Research Ethics Committee (Reference number 16/SC/0108). This study was undertaken with the local Research Ethics Committee's approval, in compliance with the Declaration of Helsinki, and with written informed consent.

### Outcome measures and potential confounders

Assessments were performed using self-completed questionnaires and in-person rating by trained researchers in outpatient clinics using standardized and validated scales, both at baseline and at each follow-up. All clinical assessments were performed in a clinically “ON” state. Motor function was assessed using the Movement Disorders Society (MDS) Unified Parkinson's Disease Rating Scale (UPDRS) part III. Part II (motor experiences of daily living) of the MDS-UPDRS was also examined as it captures both motor and non-motor aspects of the disease. Non-motor function was assessed using MDS-UPDRS part I and cognition was measured by the Montreal Cognitive Assessment (MoCA), adjusted for years of education. Mood was assessed by Hospital Anxiety and Depression Scale (HADS) and Beck Depression Inventory (BDI). Vascular risk factors, drug history including L-dopa equivalent daily dose (LEDD), and basic demographic data were also collected. For those with telephone follow-up, only MDS-UPDRS parts I and II, HADS, BDI, ESS, and RBD were made available.

### Biomarker assays

Serum samples were collected at the baseline assessment visit. The timing of the samples could not be standardized in relation to food intake. Total (TC) and HDL cholesterols (HDL-C) and albumin were measured by Abbott reagents, and fructosamine was measured by Randox reagents, all on an Abbott Architect C1600 autoanalyser. TC was measured by a linked enzymatic method, HDL-C by a chromogenic reaction after the removal of non-HDL-C, albumin by the bromocresol purple method, and fructosamine by an enzymatic assay. The assays were performed in an accredited laboratory, with 4-hourly quality control checks that showed satisfactory assay performances. Fructosamine values were corrected for albumin concentrations, according to the formula (fructosamine (mmol/L)/albumin (g/L)) x 100.^21^ Non-HDL cholesterol (non-HDL-C) was indirectly measured by subtracting HDL-C from TC, as low-density lipoprotein cholesterol cannot be calculated using non-fasting blood samples due to high triglyceride concentrations in a significant portion of them.

### Statistical analysis

We normalized our biomarkers to allow easier comparison of one biomarker compared to another. Fructosamine underwent a two-step transformation by firstly deriving a percentile rank for each value and then using the inverse-normal transformation to create normally distributed z-scores (see reference^22^ for more details) For HDL-C, we applied a natural log transformation, whereas we used a square root transformation for TC. All the biomarkers were transformed to unit standard deviation to aid direct comparison of the coefficients (Kolmogorov-Smirnov test: p = 1 (Fructosamine); p = 0.81 (HDL-C); p = 0.72 (TC)).

We used the same 4 clusters that were reported previously in the Discovery cohort,^1^ using factor analysis followed by k-means cluster analysis. The baseline clusters are characterized by (1) symmetrical motor impairment, poor olfaction, postural hypotension, and cognition; (2) mild motor and non-motor disease; (3) severe motor disease, poor sleep and psychological well-being; and (4) unilateral, tremor-dominant disease. (1) showed the fastest motor progression; (2) and (3) intermediate motor progression; (4) progressed slowest. The biomarkers were compared against the clinical clusters using multinomial logistic regression models, with the outcomes being the clinical clusters, adjusted for age, sex, and disease duration from diagnosis.

Our prognostic outcomes of interest (MDS-UPDRS parts I, II, and III, MoCA, HADS-A (anxiety), HADS-D (depression), and BDI) were modelled using longitudinal multilevel (random slope and intercept) models, with time axis being the years since diagnosis, adjusted for age at diagnosis and sex, to determine whether these biomarkers predicted disease progression. Each clinical cluster and prognosis analysis was considered separately, thus this method adjusts for the number of biomarkers analyzed. The details of this method have been published previously.^7^

Although we had a priori hypotheses, we report both p and q values for our model-based hypothesis tests to minimize any type I error. We derived q values based on a false discovery rate (FDR) method to control for multiple comparisons, and set a q < 0.05 as the significance threshold. We have tried to not use language like “significant” and “non-significant”, instead readers should view p-values as a continuum where smaller p-values represent greater evidence against the null hypothesis and confidence intervals should be examined for the strength of any association. We hope this approach will promote modern thinking that arbitrary p-value thresholds are unhelpful.^23^

### Sensitivity analyses

We adjusted the MDS-UPDRS parts II and III outcomes for the subjects’ levodopa-equivalent daily dose (LEDD), using our previously published approach (see Supplemental files of reference^7^). Simply adding treatment as a covariate in a regression model is not appropriate where treatment has a direct effect on the outcome. Instead a varying constant value, depending on the LEDD, was added to the MDS-UPDRS parts II and III totals to predict what the observed values would have been if untreated as the LEDD will mask the true value. We considered whether to adjust for statin use in our main analysis by considering two potential causal models (see Supplemental Figure 1 using directed acyclic graphs (DAGs)). In Supplemental Figure 1a, statin use is conceptualized as a potential confounder as it determines lipid levels and may predict prognosis hence should be adjusted for in a multivariable model. However evidence from the PD-STAT trial has failed to support any beneficial effect of statin use on disease progression.^24^ A more plausible causal diagram is shown in Supplemental Figure 1b where statin use is determined by prior lipid levels (unmeasured in our dataset) which in turn predicts measured baseline lipids. In this scenario statin use is an intermediary variable, hence adjustment is not appropriate as it would attenuate any true association. Further, if statins do not predict prognosis then conditioning on them could introduce “back door” confounding (illustrated by variable U in the figure) through other unadjusted covariates (e.g., health literacy) associated with both statin use and our outcomes. Hence, we decided not to include any adjustment for statin use in our main analysis but added it as a sensitivity analysis. In addition, we repeated our models excluding statin users to avoid any pleiotropic statin effect in addition to lipid lowering. Finally, we used pattern-mixture models to explore whether any of results could be biased by withdrawal from the follow-up.^25^

## Results

The descriptive data from our 866 PD patients is shown in Table 1. They had a median age at diagnosis of 67.9 years (IQR, 61.2–74.2 years), and were predominantly male (64.3%). With a median disease duration from diagnosis of 0.96 years (IQR, 0.43–1.98 years) and median follow up duration of 4.3 years (IQR, 2.7–6.0 years) from the baseline visit. The vast majority of assessments were done at our clinic with only a small percentage by telephone (141/2911; 4.8%). Overall, 271 (31.3%) were recorded as withdrawn from the study at some point.

**Table 1.** Baseline demographics of 866 PD patients.

	Mean (SD); Median (IQR) / % (n)
*Demographics*	
Age, y	67.2 (9.69); 67.9 (61.21–74.23)
Male, %	557 (64.3%)
Age at diagnosis (y)	66.0 (9.748); 66.7 (59.66–73.22)
Disease duration from diagnosis (y)	1.24 (0.947); 0.96 (0.43–1.98)
*Vascular variables*	
Heart failure	1% (9)
Angina	8.6% (74)
Myocardial infarction	4.4% (38)
Transient ischaemic attack/stroke	4.6% (40)
Vascular risk factors	
Diabetes	7.7% (67)
High cholesterol	32.1% (278)
Hypertension	34.5% (298)
Rheumatoid arthritis	0.1% (1)
BMI (kg/m^2^)	27.5 (5.013); 27.0 (24.3–29.76)
Blood pressure, average of two systolic	143.7 (19.97); 141.5 (129–156)
Orthostatic hypotension (systolic drop >20 mmHg or diastolic drop >10 mmHg)	19.5% (169)
Smoking	
Non-smoker	59.0% (505)
Ex-smoker	38.6% (330)
Current smoker	2.6% (21)
Light	1.1% (9)
Moderate	1.1% (9)
Heavy	0.4% (3)
Current coffee intake (cups per day)	
0	9.4% (81)
1	6.3% (54)
2–3	26.2% (226)
4 or more	58.1% (503)
Cardiovascular medications	40.4% (350)
Beta-blockers	12.0% (104)
ACE inhibitors	18.4% (159)
Angiotensin II and renin blockers	6.9% (60)
Diuretics	12.0% (104)
Calcium antagonists and other vasodilators	13.1% (113)
Centrally acting antihypertensives	0.4% (3)
Nitrates and other antianginals	2.5% (22)
Peripheral vasodilators	0.1% (1)
Alpha blockers	2.1% (18)
Statins	28.1% (243)
Chronic Kidney Disease	0.1% (1)
Atrial Fibrillation	2.19% (19)
*Parkinson's medications*	
L-DOPA	54.8% (475)
COMT inhibitors	1.3% (11)
Amantadine	0.7% (6)
Anticholinergics	2.0% (17)
Dopamine agonists	29.6% (256)
MAO-B inhibitors	24.8% (215)
Untreated	13.0% (113)
L-dopa equivalent daily dose (mg)	277 (210); 300 (100–400)
*Biomarker variables*	
Fructosamine (μmol/L)	238.2 (43.12); 235.9 (214.88–257.01)
Albumin (g/L)	40.3 (3.35); 40.4 (38.5–42.3)
Albumin-corrected fructosamine (μmol/g)	592.9 (105.10); 584.4 (538.15–634.25)
HDL-C (mmol/L)	1.51 (0.397); 1.47 (1.22–1.76)
Non-HDL (mmol/L)	3.34 (0.965); 3.27 (2.65–3.94)
Cholesterol (mmol/L)	4.85 (1.11); 4.84 (4.06–5.58)
Cholesterol/HDL-C ratio	3.33 (0.805); 3.23 (2.76–3.82)
*Baseline clinical data*	
MDS-UPDRS I	8.84 (5.11); 8 (5–12)
MDS-UPDRS II	8.70 (6.09); 8 (4–12)
MDS-UPDRS III	26.5 (10.91); 25 (18–34)
MDS-UPDRS IV	0.28 (1.10); 0 (0–0)
MoCA	24.9 (3.35); 25 (23–27)
HADS-A	4.54 (3.75); 4 (2–7)
HADS-D	4.39 (3.39); 4 (2–7)
BDI	8.87 (6.33); 8 (4–12)

HDL-C: high-density lipoprotein cholesterol; MDS-UPDRS: Movement Disorder Society Unified Parkinson's Disease Rating Scale; MoCA: Montreal Cognitive Assessment; HADS-A: Hospital Anxiety and Depression Scale – Anxiety; HADS-D: Hospital Anxiety and Depression Scale – Depression; BDI: Beck Depression Inventory.

At baseline, 13.0% were untreated, 54.8% were on levodopa, and 29.6% on dopamine agonists. The mean levodopa-equivalent daily dose (LEDD) at baseline was 277 mg. In terms of vascular risk factors, 59.0% were non-smokers, 7.7% had diabetes mellitus, 32.1% had hypercholesterolemia, and 34.5% had hypertension. Statins were used in 28.1% of patients. Longitudinal modelling showed deterioration of cognitive, motor function and mood with every year of increasing disease duration (p < 0.001 see Supplemental Table 1).

We found variation in HDL-C and TC levels, but not fructosamine or non-HDL-C, across our 4 PD clusters that was unlikely to be due to chance (see Table 2). This appeared to be driven by the third cluster comprising severe motor and non-motor features, which showed reduced HDL-C and TC levels, (HDL-C, p < 0.001, q < 0.001; TC, p = 0.009, q = 0.013). On adjusting for statins use, however, the effect was attenuated for cholesterol (q = 0.072, q = 0.11), though the HDL-C levels still remained lower (q < 0.001, q < 0.001, see Table 2).

**Table 2.** Biomarkers versus PD subtypes cluster with and without adjustment for statin use*.

					Without statin adjustment		With statin adjustment	
Blood biomarker	Cluster 1 n = 276	Cluster 2 n = 144	Cluster 3 n = 204	Cluster 4 n = 231	p-value	q-value	p-value	q-value
Fructosamine	603.25 (107.70)	595.75 (124.14)	591.21 (105.77)	581.06 (86.00)	0.26	0.26	0.30	0.30
HDL-C (mmol/L)	1.53 (0.38)	1.64 (0.39)	1.37 (0.37)	1.53 (0.40)	<0.001	<0.001	<0.001	<0.001
Total cholesterol (mmol/L)	4.88 (1.14)	5.10 (1.05)	4.55 (1.10)	4.91 (1.07)	0.009	0.013	0.072	0.11
Non-HDL-C	3.36 (0.98)	3.46 (0.93)	3.18 (0.99)	3.38 (0.92)	0.26	0.26	0.81	0.81

* All models were adjusted for age, gender and disease duration. Values are mean (SD). Fructosamine values have been corrected for albumin concentrations.

HDL-C: high-density lipoprotein. Clusters: (1) fast motor progression with symmetrical motor disease, poor olfaction, cognition, and postural hypotension; (2) mild motor and nonmotor disease with intermediate motor progression; (3) severe motor disease, poor psychological well-being, and poor sleep with an intermediate motor progression.

(4) slow motor progression with tremor-dominant, unilateral disease.

### Predictors of motor function and activities of daily living

Both higher HDL-C and TC, but not fructosamine, were strongly associated with better baseline MDS-UPDRS-II (intercept) even after adjusting for multiple comparisons (Table 3). However, none of these biomarkers predicted the rate of change over time (slope). Adjusting for statin use weakened the TC association but the HDL-C association remained (Supplemental Table 2). All the associations between any of the biomarkers and MDS-UPDRS part III intercept or slope were consistent with chance variability, with or without adjustment for statin use, though the patterns were similar to that seen for the ADL outcome.

**Table 3.** Associations between each cholesterol and glycaemic biomarker with motor, cognitive and mood outcome measures.

	Biomarker	Intercept (95% CI)	p-value	q-value	Slope (95% CI)	p-value	q-value
MDS- UPDRS-I	Fructosamine	−0.43 (−0.80, −0.05)	0.027	0.027	0.06 (−0.02, 0.14)	0.14	0.42
	HDL-C	−0.94 (−1.35, −0.53)	<0.001	<0.001	−0.01 (−0.09, 0.08)	0.88	0.88
	Total cholesterol	−0.47 (−0.87, −0.07)	0.021	0.027	−0.02 (−0.11, 0.06)	0.56	0.84
MDS- UPDRS-II	Fructosamine	−0.04 (−0.48, 0.40)	0.86	0.86	0.08 (−0.03, 0.18)	0.16	0.20
	HDL-C	−0.74 (−1.22, −0.26)	0.002	0.007	−0.08 (−0.19, 0.04)	0.20	0.20
	Total cholesterol	−0.51 (−0.98, −0.05)	0.031	0.047	−0.08 (−0.19, 0.04)	0.18	0.20
MDS- UPDRS-III	Fructosamine	−0.35 (−1.19, 0.49)	0.42	0.42	0.14 (−0.10, 0.37)	0.26	0.39
	HDL-C	−0.82 (−1.74, 0.10)	0.08	0.23	−0.10 (−0.35, 0.15)	0.44	0.44
	Total cholesterol	−0.66 (−1.56, 0.25)	0.15	0.23	−0.17 (−0.42, 0.08)	0.18	0.39
BDI	Fructosamine	−0.10 (−0.58, 0.38)	0.68	0.68	−0.02 (−0.12, 0.08)	0.65	0.65
	HDL-C	−0.94 (−1.45, −0.42)	<0.001	0.001	−0.07 (−0.18, 0.04)	0.21	0.31
	Total cholesterol	−0.52 (−1.03, −0.01)	0.044	0.066	−0.09 (−0.20, 0.01)	0.077	0.23
HADS-D	Fructosamine	−0.15 (−0.41, 0.11)	0.25	0.25	0.02 (−0.04, 0.07)	0.54	0.77
	HDL-C	−0.50 (−0.78, −0.22)	<0.001	0.001	0.01 (−0.05, 0.07)	0.77	0.77
	Total cholesterol	−0.28 (−0.55, −0.01)	0.046	0.068	−0.01 (−0.07, 0.04)	0.64	0.77
HADS-A	Fructosamine	−0.05 (−0.33, 0.22)	0.70	0.70	−0.00 (−0.05, 0.05)	0.92	0.95
	HDL-C	−0.35 (−0.65, −0.05)	0.021	0.054	0.00 (−0.05, 0.06)	0.90	0.95
	Total cholesterol	−0.31 (−0.60, −0.02)	0.036	0.054	0.00 (−0.05, 0.06)	0.95	0.95
MoCA	Fructosamine	0.17 (−0.06, 0.40)	0.15	0.15	−0.04 (−0.10, 0.02)	0.17	0.36
	HDL-C	0.33 (0.08, 0.58)	0.010	0.014	−0.04 (−0.10, 0.02)	0.24	0.36
	Total cholesterol	0.34 (0.10, 0.59)	0.006	0.014	−0.01 (−0.07, 0.05)	0.67	0.67

HDL-C: high-density lipoprotein cholesterol; MDS-UPDR:, Movement Disorder Society Unified Parkinson's Disease Rating Scale; MoCA: Montreal Cognitive Assessment; HADS-A: Hospital Anxiety and Depression Scale – Anxiety; HADS-D: Hospital Anxiety and Depression Scale – Depression; BDI: Beck Depression Inventory.

Model adjusted for age and sex. Coefficients for a standardized change in biomarker.

### Predictors of non-motor function

We also found that higher HDL-C, TC and fructosamine were associated with better baseline MDS-UPDRS-I (Table 3), although the association with TC was lost after adjustment for statin use (Supplemental Table 2). None of these biomarkers predicted the longitudinal change in UPDRS-I. Raised HDL-C and TC, though not fructosamine, were associated with better mood scores on HADS-D, HADS-A and BDI at baseline. No biomarker predicted the longitudinal slope for either depression or anxiety. Raised HDL-C and TC, though not fructosamine, were associated with better baseline general cognition, even after adjusting for multiple testing and statin use, but none of the biomarkers predicted the change in MoCA over time.

### Sensitivity analyses

The results from our sensitivity analyses using pattern-mixture models to account for withdrawal (Supplemental Table 3) and adjustment for LEDD (Supplemental Table 4) were similar to those from the main analyses, although the association between TC and UPDRS-II intercept was consistent with chance in the pattern-mixture model (p = 0.09, q = 0.13). On adjustment for withdrawal, there was a weak association between HDL-C and UPDRS-II slope (p = 0.05), with a 1 SD increase being associated with a 0.12 decrease in UPDRS-II change per year. Hence, a higher HDL-C showed a weak association with a better ADL prognosis. After adjusting for multiple comparisons there was little evidence against the null hypothesis (q = 0.08). Similarly, there was also a modest association between HDL-C and BDI (p = 0.03), with a 1 SD increase being associated with a 0.13 decrease in BDI change per year. This was again weakened after adjusting for multiple comparisons (q = 0.08). A sub-group analysis excluding around a third of the patients who were on statins (Supplemental Table 5) found the same patterns for HDL-C, but weak and inverse associations with TC except for UPDRS-III. A higher TC predicted a slower increase in BDI over time, but this may have been a chance finding (p = 0.04, q = 0.08).

## Discussion

Some but not all of our *a priori* hypotheses were supported in the Discovery cohort, one of the largest and best phenotyped studies of the natural history of PD. Our findings for TC are consistent with past evidence in that elevated levels were associated with a less severe cluster at presentation (cluster 2) and higher levels predicted better ADL functioning, psychological well-being and cognitive function at baseline, though the associations with better motor function were consistent with chance. HDL-C showed very similar patterns of results to TC and the effects appeared more pronounced, except for MOCA. Raised HDL-C showed stronger inverse associations with part II than part III of the UPDRS score, though both are supposed to capture motor function. It is possible that this reflects the impact of mood and cognition on real world motor function measured by part II as higher HDL-C was also associated with better profiles for these outcomes. None of the biomarkers were predictive of future rate of change though obviously subjects with more severe disease early in the natural history will still be worse than their peers over the subsequent years as there was no evidence of convergence in scores. In general, adjustment for statin use attenuated the associations with TC, though this may have been due to over-adjustment. Our sub-group analysis of non-statin users supported these results but was less powered due to the smaller sample size.

In contrast, we found no real associations with levels of fructosamine except for an inverse association with UPDRS-I at baseline so that higher fructosamine was associated with better, not worse, scores, counter to our a priori and may be due to a type I error.

While HDL-C has been shown to be protective in cardiovascular diseases, it may also mediate neuroprotective effects through Apo A-I, which has been shown to be associated with greater putaminal dopamine transporter (DAT) deficit on imaging, in the prodromal compared to manifest phases of PD.^26^ Statins may or may not have a protective effect in PD, though results from the recent PD-STAT phase II trial failed to find evidence of any clinically important slowing in the rate of change in motor function (UPDRS-III), similar to our lack of associations with TC on longitudinal outcomes.^24^

Studies in non-PD patients with major depressive disorder have shown reduced TC levels and reduced HDL-C levels when compared to controls, especially in patients ≥40 years-old.^27^ As cholesterol is integral to formation of synaptosomal membranes, reduced levels may decrease serotonin receptors in the brain. A recent small-scale study has also shown reduced Lp(a) levels, which is structurally similar to LDL-C, in PD patients correlated with higher HAMD scores for depression.^28^ However, our preliminary findings that non-HDL-C, which includes other proatherogenic lipoproteins including LDL-C, was not associated with any mood variable intercepts at baseline, suggests other mechanisms behind effects of TC on mood. The effect of HDL-C and cholesterol on dopamine receptors may have similar effects in other neurotransmitter pathways in a multisystem degenerative disease like PD.^29^

Our study has detailed phenotypic data collected longitudinally on a relatively large sample, and has allowed us to detect moderate sized associations with increased statistical power in comparison to previous progression studies that have been limited in size, only have measures from cases with advanced disease or do not measure disease phenotype directly.^4,12,30^ We used sophisticated statistical methods to simultaneously model the baseline and trajectories of several important PD outcomes including motor and non-motor domains. We also accounted for statin therapy as well as potential selection bias through loss to follow-up.

There are still several limitations to our study that need to be considered. We examined multiple biomarkers with multiple outcomes over time. One must be careful in interpreting statistical significance due to type I errors, even though we corrected for multiple comparisons and provide effect estimates and 95% confidence intervals. As only baseline samples were available, we were unable to assess longitudinal changes in the analytes and their associations with clinical indices. However, very large changes in lipid profiles tend not to occur over a few years unless patients are instituted on lipid-modifying medications (a possibility with some participants in this study) or undertake a significant change in their diet. With continuous progression of the disease, there may be more severe impairment in movement, body weight, appetite, and sympathetic activity that may lead to changes in serum levels of these biomarkers and subsequent progression of the disease. Unfortunately, we do not have high quality data on diet and physical activity to describe this. However, the baseline measures are less likely to reflect reverse causation secondary to disease severity. Our study is observational and therefore one cannot assume that any associations we observe would also occur after an intervention such as medication. It is important to note that there is a significant prodromal period before the diagnosis of PD and significant changes in serum metabolomic profile may have already occurred. It would be interesting to investigate longitudinal changes of serum biomarkers in isolated REM sleep behavior disorder patients before their subsequent progression to PD. Since fasting serum specimens were unavailable for measuring triglycerides and thereby calculating LDL-C, our association analyses were limited to, and based on, total, HDL and non-HDL cholesterols. Non-HDL cholesterol has been shown to correlate well with LDL-C, particularly small dense LDL-C, which can penetrate endothelium better, and, especially when oxidized, promote pathogenicities including α-synucleinopathies.^31^ Although serum fructosamine may not be as good a marker of glucose status as HbA_1c_, it has been shown to correlate well with glucose status and HbA_1c_ levels.^32^ Like any longitudinal study we had missing data especially due to cohort attrition. Our multi-level models will have accounted for missingness under the “missing at random” assumption and our sensitivity analyses using “pattern-mixture” models suggest that no major biases were introduced because of loss to follow-up.

In conclusion we have found that a higher HDL-C and to a lesser degree TC are associated with a better PD phenotype that encompasses both motor and non-motor features though neither measure predicted a faster or slower trajectory in motor and non-motor functions. Fructosamine was not associated with phenotypic differences. Further work is required to assess whether these metabolic changes precede PD diagnosis and are also found in the prodromal period to establish if they may have a primary causal role in disease phenotype.

## Supplemental Material

sj-docx-1-pkn-10.1177_1877718X251323914 - Supplemental material for Association of cholesterol and glycemic state biomarkers with phenotypic variation and Parkinson's disease progression: The Oxford Discovery cohort Supplemental material, sj-docx-1-pkn-10.1177_1877718X251323914 for Association of cholesterol and glycemic state biomarkers with phenotypic variation and Parkinson's disease progression: The Oxford Discovery cohort by Nicholas Kwan Ho Chiu, Michael Lawton, Tanja Zerenner, Alireza Morovat, Jessica Welch, Jamil Razzaque, Michele T Hu and Yoav Ben-Shlomo in Journal of Parkinson's Disease
