# Supplementary material for: Association of cholesterol and glycemic state biomarkers with phenotypic variation and Parkinson's disease progression: The Oxford Discovery cohort
Source: J Parkinsons Dis. 2025 Apr 13:1877718X251323914. Online ahead of print. doi: 10.1177/1877718X251323914 (PMC13347451; doi:10.1177/1877718X251323914)
Supplement: Supplementary file 1 [file sj-docx-1-pkn-10.1177_1877718X251323914.docx]

**Supplemental Material**

**Association of cholesterol and glycemic state biomarkers with phenotypic variation and Parkinson’s disease progression: The Oxford Discovery cohort**

**Supplemental Figure 1.** DAGs: Statin use as potential confounder (a) or as intermediary variable (b)

(a) **(b)**

U indicates potential unmeasured confounders in the association between statins use and prognosis.

**Supplemental Table 1.** Associations between motor, cognitive, and mood outcome measures with disease duration

|  | Intercept (95% CI) | p | Slope (95% CI) | p |
| --- | --- | --- | --- | --- |
| MDS-UPDRS I | 7.93 (7.56, 8.30) | <0.001 | 0.74 (0.66, 0.82) | <0.001 |
| MDS-UPDRS II | 6.85 (6.42, 7.28) | <0.001 | 1.36 (1.25, 1.47) | <0.001 |
| MDS-UPDRS III | 23.47 (22.64, 24.30) | <0.001 | 2.37 (2.13, 2.61) | <0.001 |
| MoCA | 24.94 (24.70, 25.18) | <0.001 | -0.12 (-0.18, -0.07) | <0.001 |
| HADS-A | 4.24 (3.96, 4.51) | <0.001 | 0.16 (0.11, 0.21) | <0.001 |
| HADS-D | 4.03 (3.78, 4.28) | <0.001 | 0.22 (0.16, 0.27) | <0.001 |
| BDI | 8.20 (7.74, 8.68) | <0.001 | 0.63 (0.53, 0.73) | <0.001 |

Longitudinal multilevel model including only disease duration as predictor (fixed and random effect). HDL-C: high-density lipoprotein cholesterol; MDS-UPDRS: Movement Disorder Society Unified Parkinson’s Disease Rating Scale; MoCA: Montreal Cognitive Assessment; HADS-A: Hospital Anxiety and Depression Scale – Anxiety; HADS-D: Hospital Anxiety and Depression Scale – Depression; BDI: Beck Depression Inventory.

**Supplemental Table 2.** Associations between each cholesterol and glycaemia biomarker and motor, cognitive and mood outcome measures adjusted for statin use

|  | Biomarker | Intercept (95% CI) | p | q | Slope (95% CI) | p | q |
| --- | --- | --- | --- | --- | --- | --- | --- |
| MDS- UPDRS-I | Fructosamine | -0.41 (-0.79, -0.04) | **0.031** | **0.047** | 0.06 (-0.02, 0.14) | 0.14 | 0.38 |
|  | HDL-C | -0.89 (-1.30, -0.48) | **<0.001** | **<0.001** | -0.01 (-0.10, 0.07) | 0.78 | 0.78 |
|  | Total Cholesterol | -0.34 (-0.80, 0.12) | 0.14 | 0.14 | -0.06 (-0.15, 0.04) | 0.25 | 0.38 |
| MDS- UPDRS-II | Fructosamine | -0.03 (-0.47, 0.41) | 0.89 | 0.89 | 0.08 (-0.03, 0.18) | 0.16 | 0.18 |
|  | HDL-C | -0.71 (-1.20, -0.22) | **0.004** | **0.012** | -0.08 (-0.20, 0.04) | 0.18 | 0.18 |
|  | Total Cholesterol | -0.48 (-1.02, 0.06) | 0.080 | 0.12 | -0.11 (-0.23, 0.02) | 0.10 | 0.18 |
| MDS- UPDRS-III | Fructosamine | -0.33 (-1.17, 0.51) | 0.44 | 0.44 | 0.13 (-0.10, 0.37) | 0.26 | 0.56 |
|  | HDL-C | -0.82 (-1.75, 0.11) | 0.083 | 0.20 | -0.08 (-0.33, 0.18) | 0.56 | 0.56 |
|  | Total Cholesterol | -0.80 (-1.85, 0.25) | 0.13 | 0.20 | -0.10 (-0.39, 0.19) | 0.49 | 0.56 |
| BDI | Fructosamine | -0.08 (-0.56, 0.40) | 0.74 | 0.74 | -0.02 (-0.12, 0.08) | 0.64 | 0.64 |
|  | HDL-C | -0.86 (-1.38, -0.33) | **0.001** | **0.004** | -0.07 (-0.18, 0.04) | 0.19 | 0.29 |
|  | Total Cholesterol | -0.27 (-0.85, 0.31) | 0.36 | 0.54 | -0.12 (-0.24, -0.01) | **0.036** | 0.11 |
| HADS-D | Fructosamine | -0.14 (-0.40, 0.12) | 0.28 | 0.34 | 0.02 (-0.04, 0.07) | 0.55 | 0.83 |
|  | HDL-C | -0.46 (-0.74, -0.18) | **0.001** | **0.004** | 0.00 (-0.06, 0.06) | 0.97 | 0.97 |
|  | Total Cholesterol | -0.15 (-0.46, 0.16) | 0.34 | 0.34 | -0.05 (-0.11, 0.02) | 0.14 | 0.41 |
| HADS-A | Fructosamine | -0.05 (-0.32, 0.23) | 0.74 | 0.74 | -0.00 (-0.05, 0.05) | 0.91 | 0.96 |
|  | HDL-C | -0.32 (-0.62, -0.01) | **0.041** | 0.12 | -0.00 (-0.06, 0.05) | 0.96 | 0.96 |
|  | Cholesterol | -0.22 (-0.55, 0.12) | 0.20 | 0.30 | -0.02 (-0.08, 0.04) | 0.55 | 0.96 |
| MoCA | Fructosamine | 0.16 (-0.07, 0.39) | 0.17 | 0.17 | -0.04 (-0.09, 0.02) | 0.17 | 0.38 |
|  | HDL-C | 0.30 (0.05, 0.55) | 0.020 | 0.060 | -0.03 (-0.09, 0.03) | 0.26 | 0.38 |
|  | Total Cholesterol | 0.29 (0.00, 0.57) | 0.047 | 0.070 | -0.01 (-0.08, 0.06) | 0.75 | 0.75 |

HDL-C: high-density lipoprotein cholesterol; MDS-UPDRS: Movement Disorder Society Unified Parkinson’s Disease Rating Scale; MoCA: Montreal Cognitive Assessment; HADS-A: Hospital Anxiety and Depression Scale – Anxiety; HADS-D: Hospital Anxiety and Depression Scale – Depression; BDI: Beck Depression Inventory.

Model adjusted for age, sex and statin use. Coefficients for a standardized change in biomarker

**Supplemental Table 3.** Pattern Mixture Model (adjustment for withdrawal) longitudinal follow-up associations (per standard deviation change in biomarkers).

|  | Biomarker | Intercept (95% CI) | p | q | Slope (95% CI) | p | q |
| --- | --- | --- | --- | --- | --- | --- | --- |
| MDS- UPDRS-I | Fructosamine | -0.47 (-0.85, -0.09) | **0.016** | **0.022** | 0.07 (-0.01, 0.16) | 0.079 | 0.24 |
|  | HDL-C | -0.89 (-1.30, -0.47) | **<0.001** | **<0.001** | -0.04 (-0.14, 0.05) | 0.36 | 0.55 |
|  | Total Cholesterol | -0.48 (-0.88, -0.07) | **0.022** | **0.022** | -0.01 (-0.10, 0.08) | 0.85 | 0.85 |
| MDS- UPDRS-II | Fructosamine | -0.12 (-0.57, 0.32) | 0.59 | 0.59 | 0.12 (0.01, 0.22) | **0.033** | 0.076 |
|  | HDL-C | -0.67 (-1.16, -0.18) | **0.007** | **0.021** | -0.12 (-0.24, 0.00) | **0.050** | 0.076 |
|  | Total Cholesterol | -0.41 (-0.89, 0.06) | 0.089 | 0.13 | -0.08 (-0.20, 0.03) | 0.17 | 0.17 |
| MDS- UPDRS-III | Fructosamine | -0.49 (-1.34, 0.36) | 0.26 | 0.26 | 0.21 (-0.03, 0.46) | 0.089 | 0.27 |
|  | HDL-C | -0.85 (-1.79, 0.09) | 0.076 | 0.23 | -0.07 (-0.35, 0.20) | 0.60 | 0.60 |
|  | Total Cholesterol | -0.64 (-1.57, 0.28) | 0.17 | 0.26 | -0.14 (-0.40, 0.13) | 0.32 | 0.48 |
| BDI | Fructosamine | -0.08 (-0.57, 0.41) | 0.74 | 0.74 | -0.04 (-0.15, 0.06) | 0.42 | 0.42 |
|  | HDL-C | -0.85 (-1.38, -0.33) | **0.002** | **0.005** | -0.13 (-0.25, -0.01) | **0.028** | 0.084 |
|  | Total Cholesterol | -0.50 (-1.02, 0.02) | 0.059 | 0.089 | -0.09 (-0.20, 0.02) | 0.12 | 0.19 |
| HADS-D | Fructosamine | -0.15 (-0.41, 0.11) | 0.26 | 0.26 | 0.01 (-0.05, 0.07) | 0.68 | 0.96 |
|  | HDL-C | -0.50 (-0.78, -0.21) | **<0.001** | **0.002** | 0.00 (-0.06, 0.07) | 0.96 | 0.96 |
|  | Total Cholesterol | -0.32 (-0.59, -0.04) | **0.025** | **0.038** | 0.02 (-0.04, 0.08) | 0.56 | 0.96 |
| HADS-A | Fructosamine | -0.04 (-0.32, 0.24) | 0.78 | 0.78 | -0.01 (-0.06, 0.05) | 0.78 | 0.78 |
|  | HDL-C | -0.30 (-0.60, 0.00) | 0.053 | 0.079 | -0.03 (-0.09, 0.04) | 0.42 | 0.78 |
|  | Cholesterol | -0.32 (-0.62, -0.02) | **0.034** | 0.079 | 0.01 (-0.05, 0.07) | 0.72 | 0.78 |
| MoCA | Fructosamine | 0.21 (-0.02, 0.45) | 0.074 | 0.074 | -0.06 (-0.12, 0.00) | 0.054 | 0.16 |
|  | HDL-C | 0.33 (0.07, 0.59) | **0.011** | **0.034** | -0.04 (-0.10, 0.03) | 0.29 | 0.43 |
|  | Total Cholesterol | 0.29 (0.03, 0.54) | **0.026** | **0.040** | -0.00 (-0.07, 0.06) | 0.89 | 0.89 |

HDL-C: high-density lipoprotein cholesterol; MDS-UPDRS: Movement Disorder Society Unified Parkinson’s Disease Rating Scale; MoCA: Montreal Cognitive Assessment; HADS-A: Hospital Anxiety and Depression Scale – Anxiety; HADS-D: Hospital Anxiety and Depression Scale – Depression; BDI: Beck Depression Inventory.

Model adjusted for age and sex. Coefficients for a standardized change in biomarker

**Supplemental Table 4.** Associations between lipid and glucose biomarkers and MDS-UPDRS II and III, adjusted for levodopa equivalent daily dose

|  | Biomarker | Intercept (95% CI) | p | q | Slope (95% CI) | p | q |
| --- | --- | --- | --- | --- | --- | --- | --- |
| MDS- UPDRS-II | Fructosamine | -0.17 (-0.63, 0.29) | 0.46 | 0.46 | 0.09 (-0.02, 0.19) | 0.12 | 0.33 |
|  | HDL-C | -0.79 (-1.29, -0.29) | **0.002** | **0.006** | -0.05 (-0.17, 0.07) | 0.41 | 0.41 |
|  | Total Cholesterol | -0.50 (-0.99, -0.02) | **0.043** | 0.065 | -0.07 (-0.19, 0.04) | 0.22 | 0.33 |
|  |  |  |  |  |  |  |  |
| MDS- UPDRS-III | Fructosamine | -0.66 (-1.51, 0.20) | 0.13 | 0.18 | 0.15 (-0.09, 0.39) | 0.21 | 0.31 |
|  | HDL-C | -0.88 (-1.82, 0.05) | 0.064 | 0.18 | -0.09 (-0.35, 0.16) | 0.48 | 0.48 |
|  | Total Cholesterol | -0.62 (-1.54, 0.30) | 0.18 | 0.18 | -0.17 (-0.43, 0.08) | 0.18 | 0.31 |

HDL-C: high-density lipoprotein cholesterol; MDS-UPDRS: Movement Disorder Society Unified Parkinson’s Disease Rating Scale;

Model adjusted for age and sex. Coefficients for a standardized change in biomarker.

**Supplemental Table 5.** Associations between lipid and glucose biomarkers and motor, cognitive and mood outcome measures for PD patients not on statins

|  | Biomarker | Intercept (95% CI) | p | q | Slope (95% CI) | p | q |
| --- | --- | --- | --- | --- | --- | --- | --- |
| MDS- UPDRS-I | Fructosamine | -0.41 (-0.84, 0.03) | 0.067 | 0.10 | 0.07 (-0.02, 0.17) | 0.12 | 0.37 |
|  | HDL-C | -0.92 (-1.38, -0.46) | **< 0.001** | **<0.001** | 0.02 (-0.08, 0.12) | 0.69 | 0.69 |
|  | Total Cholesterol | -0.16 (-0.67, 0.35) | 0.55 | 0.55 | -0.04 (-0.15, 0.07) | 0.49 | 0.69 |
| MDS- UPDRS-II | Fructosamine | -0.12 (-0.65, 0.41) | 0.66 | 0.66 | 0.06 (-0.06, 0.18) | 0.34 | 0.48 |
|  | HDL-C | -0.77 (-1.34, -0.20) | **0.008** | **0.024** | -0.05 (-0.19, 0.09) | 0.48 | 0.48 |
|  | Total Cholesterol | -0.35 (-0.97, 0.27) | 0.27 | 0.41 | -0.10 (-0.25, 0.05) | 0.17 | 0.48 |
| MDS- UPDRS-III | Fructosamine | -0.01 (-1.09, 1.06) | 0.98 | 0.98 | 0.15 (-0.14, 0.44) | 0.31 | 0.79 |
|  | HDL-C | -0.88 (-2.03, 0.27) | 0.13 | 0.20 | -0.06 (-0.37, 0.25) | 0.70 | 0.79 |
|  | Total Cholesterol | -1.03 (-2.30, 0.23) | 0.11 | 0.20 | -0.05 (-0.39, 0.29) | 0.79 | 0.79 |
| BDI | Fructosamine | -0.29 (-0.83, 0.26) | 0.30 | 0.45 | 0.01 (-0.10, 0.13) | 0.83 | 0.83 |
|  | HDL-C | -0.63 (-1.22, -0.04) | **0.035** | 0.10 | -0.06 (-0.19, 0.06) | 0.31 | 0.47 |
|  | Total Cholesterol | -0.03 (-0.66, 0.61) | 0.94 | 0.94 | -0.15 (-0.28, -0.02) | **0.028** | 0.084 |
| HADS-D | Fructosamine | -0.28 (-0.58, 0.03) | 0.080 | 0.12 | 0.05 (-0.02, 0.11) | 0.17 | 0.27 |
|  | HDL-C | -0.42 (-0.75, -0.09) | **0.013** | **0.039** | 0.01 (-0.06, 0.08) | 0.78 | 0.78 |
|  | Total Cholesterol | 0.00 (-0.36, 0.36) | 0.99 | 0.99 | -0.05 (-0.13, 0.02) | 0.18 | 0.27 |
| HADS-A | Fructosamine | -0.01 (-0.35, 0.33) | 0.94 | 0.94 | -0.00 (-0.06, 0.06) | 0.90 | 0.90 |
|  | HDL-C | -0.24 (-0.60, 0.12) | 0.19 | 0.49 | 0.01 (-0.06, 0.07) | 0.83 | 0.90 |
|  | Cholesterol | -0.19 (-0.58, 0.19); | 0.33 | 0.49 | -0.02 (-0.09, 0.05) | 0.60 | 0.90 |
| MoCA | Fructosamine | 0.18 (-0.11, 0.46) | 0.22 | 0.33 | -0.04 (-0.10, 0.03) | 0.28 | 0.52 |
|  | HDL-C | 0.23 (-0.07, 0.53) | 0.14 | 0.33 | -0.03 (-0.10, 0.04) | 0.35 | 0.52 |
|  | Total Cholesterol | 0.13 (-0.19, 0.46) | 0.42 | 0.42 | -0.00 (-0.08, 0.07) | 0.90 | 0.90 |

HDL-C: high-density lipoprotein cholesterol; MDS-UPDRS: Movement Disorder Society Unified Parkinson’s Disease Rating Scale;

Model adjusted for age and sex. Coefficients for a standardized change in biomarker
